# ACLMHA and FML: A brain-inspired kinship verification framework

**DOI:** 10.3389/fnins.2022.1093071

**Published:** 2022-12-12

**Authors:** Chen Li, Menghan Bai, Lipei Zhang, Ke Xiao, Wei Song, Hui Zeng

**Affiliations:** ^1^School of Information, North China University of Technology, Beijing, China; ^2^Shunde Innovation School, University of Science and Technology Beijing, Foshan, China

**Keywords:** brain-inspired, relation comparison network, multi-head attention, facial kinship verification, deep learning

## Abstract

As an extended research direction of face recognition, kinship verification based on the face image is an interesting yet challenging task, which aims to determine whether two individuals are kin-related based on their facial images. Face image-based kinship verification benefits many applications in real life, including: missing children search, family photo classification, kinship information mining, family privacy protection, etc. Studies presented thus far provide evidence that face kinship verification still offers many challenges. Hence in this paper, we propose a novel kinship verification architecture, the main contributions of which are as follows: To boost the deep model to capture various and abundant local features from different local face regions, we propose an attention center learning guided multi-head attention mechanism to supervise the learning of attention weights and make different attention heads notice the characteristics of different regions. To combat the misclassification caused by single feature center loss, we propose a family-level multi-center loss to ensure a more proper intra/inter-class distance measurement for kinship verification. To measure the potential similarity of features among relatives better, we propose to introduce the relation comparison module to measure the similarity among features at a deeper level. Extensive experiments are conducted on the widely used kinship verification dataset—Family in the Wild (FIW) dataset. Compared with other state-of-art (SOTA) methods, encouraging results are obtained, which verify the effectiveness of our proposed method.

## Introduction

As an extended and novel research branch of face recognition, kinship verification has received an increasing amount of attention (Hu et al., 2017; Lu et al., 2017; Wu et al., 2018; Dahan and Keller, 2020) in the recent 10 years. The purpose of kinship verification is to offer verdict whether people with different identities have kinship or not based on their facial information. Face image-based kinship verification benefits many applications in real life, including: kinship information mining (Robinson et al., 2021), missing children search (Robinson et al., 2020), family photo classification (Xia et al., 2012), family privacy protection (Kumar et al., 2020), etc. Generally, kinship can be divided into three generations containing 11 types. The same-generation: Brother-Brother (B-B), Sister-Sister (S-S), and Brother-Sister (SIBS). The first-generation: Father-Son (F-S), Father-Daughter (F-D), Mother-Son (M-S), and Mother-Daughter (M-D). The second-generation: Grandfather-Grandson (GF-GS), Grandfather-Granddaughter (GF-GD), Grandmother-Grandson (GM-GS), and Grandmother-Granddaughter (GM-GD).

**Figure 1:**
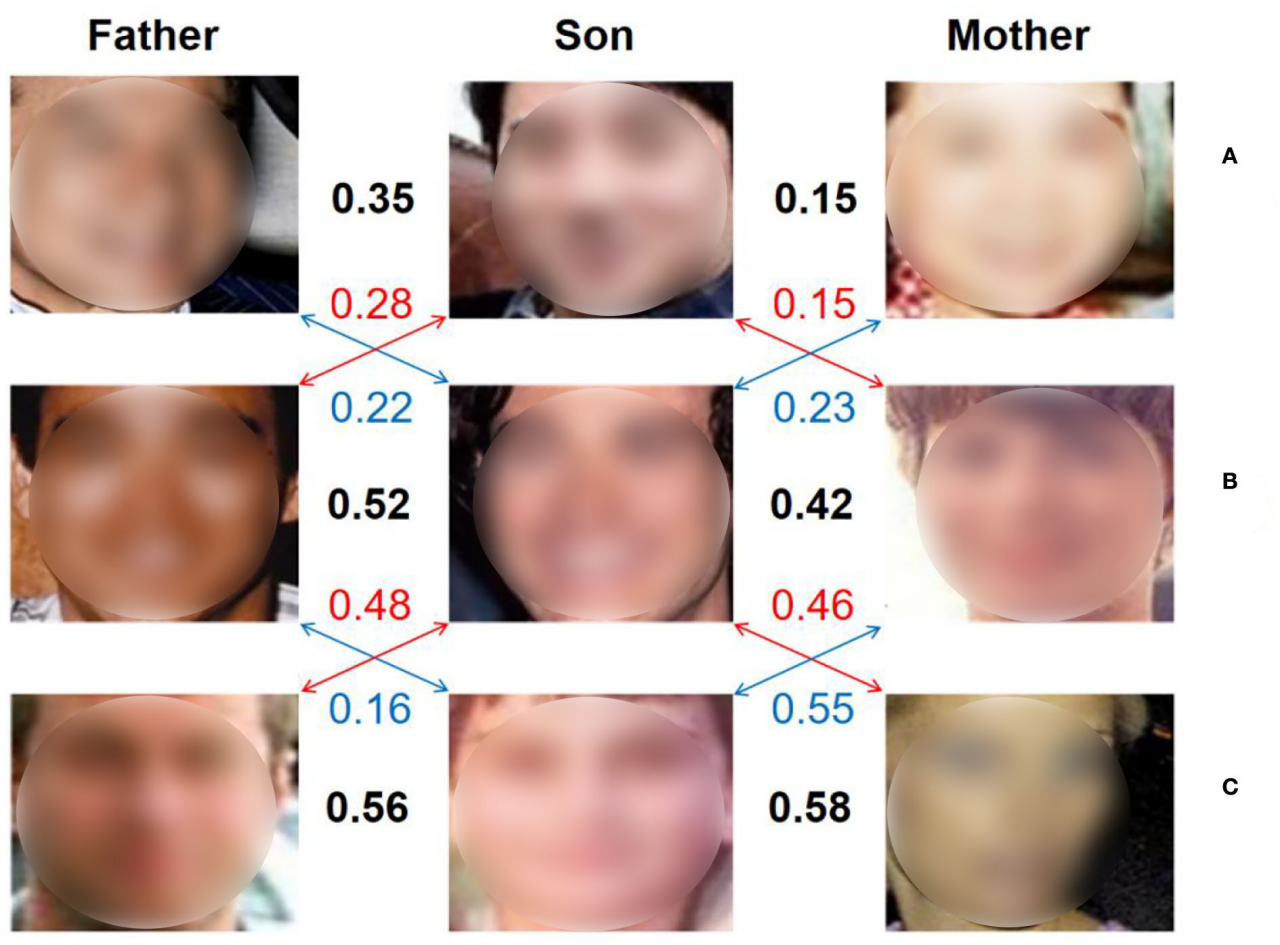
**(A–C)** The figure above shows the face features extracted by using the pretrained FaceNet (Schroff et al., 2015), and the similarity is calculated.

Meaningful achievement in kinship verification has been delivered. The earliest solution toward kinship verification is to construct proper handcraft features and then to calculate the similarity between features to verify the kinship of two face images. In recent years, with the development of deep learning which draws inspiration from the neurobiological mechanisms of the human brain, many data-driven kinship verification methods based on deep learning have been applied to solve the problem of face kinship verification. However, the achievements in kinship verification are relatively less inspirational compared to general face recognition or verification, due to the following challenges put forth:

1. Face datasets with family relationships are scarce. The scale of kinship verification dataset is incomparable to that of the general face recognition dataset. Therefore, data deficiency and imbalance invalidate many data-driven methods and pose great challenges for kinship verification. It is still very challenging to tackle the issue of how to boost its verification ability through limited data like the human brain.
2. Feature expressions of the latent similarity among family members are quite different compared to that of a single individual. To illustrate this issue, nine face images from Family in the Wild (FIW) dataset (Robinson et al., 2018) are shown in Figure 1, in which, faces in line A, line B, and line C belongs to three different families. And the first column are faces of fathers, the second column are faces of sons, the third column are faces of mothers. The similarities among those faces are calculated by adopting features extracted by the pretrained FaceNet. The face images in Figure 1A are those of a couple and their son. Due to gender differences, the calculated similarity between the son and his mother is lower than the similarity between him and fathers from other families, which is quite different with the human brain. Similarly, due to the differences in skin color, in group B, the faces of the son and the other father with white skin are also very similar. Therefore, feature expression for kinship verification is still very challenging.

Measurement of feature distance for kinship verification is much more complicated, compared to general face recognition. The main idea behind the face recognition problem is to reduce the intra-class distance between different samples of each individual and to expand the inter-class distance between samples of different individuals. However, deep learning-based models cannot handle the validation problem across multiple samples as well as the human brain, because there are usually gender differences and there is a large age gap lying between the relative samples, which make it very difficult to narrow down the intra-class distance with general hand-designed metric functions. Besides, a family usually contains several members with different feature representations. Simply adopting a single center for all the different family members generates an improper intra/inter-class distance for kinship verification. For instance: inter-distance between husband and wife is closer than their intra-class distance, which leads to a wrong verification of kinship.

To address these challenges in kinship verification, we propose an efficient and practical automatic kinship verification architecture inspired by the perspective of the human brain in processing visual information about relatives. The main contributions toward this article are as follows:

1. To boost the deep model to capture various and abundant local features from different local face regions, we propose an attention center learning guided multi-head attention mechanism to supervise the learning of attention weights and make different attention heads notice the characteristics of different regions. And then, the captured local features are combined with the global feature as the final feature expression.
2. To combat the misclassification caused by single feature center, we propose a family-level multi-center loss to ensure that the learned model can map different facial features of individuals with kinship to similar positions in the feature space.
3. To measure the potential similarity of features among relatives better, we propose to introduce the relation comparison module to measure the relationship between features at a deeper level, instead of using a hand-designed metric function.

The rest of the article is organized as follows: In section Related work, recent influential works on kinship verification are reviewed. In section Methodology, the proposed novel methods are elaborated. In section Experimental results, extensive experiments are conducted and experimental results demonstrate that our approach achieves state-of-the-art results compared to other methods. Lastly, we summarize the main ideas and contributions of this paper.

## Related work

According to the challenges discussed before, kinship verification methods based on facial features are roughly divided into local feature-based methods and metric learning-based methods.

For local feature-based methods, the key issue one needs to solve is how to abstract discriminative local features. In Zhang et al. (2015), the face image is cropped into multiple overlapping patches, and then shallow convolutional networks are used to learn the local features between relatives' face pairs for kinship verification. Dibeklioglu (2017) proposes an Age Uniform Network (AUN) to convert faces of relatives into the same age range for reducing the age features learned by the Verification Network (VFN) to be intrusive. In a similar work, a variety of artificially designed feature descriptors and deep network feature descriptors are used to extract the local and global features of the face, and the two tasks of face verification and kinship verification are combined to improve the accuracy of kinship verification (Kohli et al., 2016). In Zhang et al. (2020), adversarial loss and verification loss are added to the feature extraction process of face patches to learn the potential features of relatives' faces. Local features are used to enhance global features, which result in more effective features for kinship verification. By combining the face identification network and the face landmark prediction network, the extraction of facial appearance features and shape features is completed, and then the comparison scores of these two features are combined to finally obtain the kinship verification score (Zhang et al., 2019). In addition, in Goyal and Meenpal (2020), Dual-Tree Complex Wavelet Transform (DTCWT) is used to select a more effective patch pair for kinship verification, so as to make full use of the face patch to improve the effect of kinship verification. In Zheng et al. (2021), Residual Factorization Module is used to decompose facial features into identity and gender features, and then the adversarial training is used to reduce the negative impact of gender features on kinship verification. Indeed, local features have a positive effect on the verification of facial kinship. However, most existing local feature extraction methods rely heavily on the accuracy of facial patch crop or face landmark prediction.

To address this issue further, researchers began to introduce a non-local attention module as a complement. Visual attention is a subjective or objective mechanism of visual information selection by the brain, which concentrates on a limited amount of information and ignores other perceivable information (Cohen et al., 2012; Wang et al., 2022). It allows the human brain to be selective in processing visual input from the outside world (Yarbus, 2013), as well as enables the brain to quickly extract different parts of interest from complex scenes and process them separately (Higgins et al., 2021). Aiming to simulate the information processing mechanism of the human brain, researchers have widely discussed the topic of attention in deep neural network. Non-local attention (Wang et al., 2018) is an attention mechanism that captures the relationship between distant pixels. After fusing the information of the global feature, an autocorrelation matrix is generated to weigh the original feature map to obtain the final attention feature. This attention model represents the importance of local regions better. Many improvements have been made on the basis of a non-local attention mechanism. DANet (Dual Attention Network) (Fu et al., 2019) increases channel attention on the basis of spatial attention in non-local attention. CCNet (Criss-Cross Network) (Huang et al., 2019) uses the cross multiplication method to reduce the computation of non-local attention. OCNet (Object Contact Network) (Yuan et al., 2018) is used to obtain the pixel-level similarity of different objects in the image to obtain the target semantic information in the image better. ABD-Net (Attentive but Diverse Network) (Chen et al., 2019) uses a non-local attention model with channel and spatial attention in person re-identification model to enhance the effectiveness of local features. It has been proven that non-local attention has a good effect in extracting the importance of the image region. NLA-FFNet (Non-local Attentional Feature Fusion Network) (Zhou et al., 2022) is proposed to enhance the robustness of feature extraction by representing the relationship between features with non-local attention through a multi-layer non-local attention mechanism. At the same time (Fu et al., 2017; Zheng et al., 2017), have shown that different channels of image features can represent specific visual patterns, and grouping them can get different regions in the image. Due to the powerful feature representation capability for Siamese models with shared weights, the Siamese networks have been used by scholars to extract global features of different images (Han et al., 2022). At present, how to boost the attention module to capture various and abundant local features from different local face regions automatically as the human brain and how to make full use of local as well as global features are worth being discussed.

Metric learning has shown a promising performance for face verification and face recognition task, which provides a positive inspiration for kinship verification. In Feng and Ma (2021), a contrastive loss function suitable for kinship comparison is proposed, and a dual-path autoencoder network is used to generate another member of the family to verify kinship pairs. In Li et al. (2017), a sampling strategy of face image triples based on family relationship information is proposed. Compared with triples based on family tags, this method is more suitable for optimization on family relationship data. At the same time, there are also studies using quadruple sampling to find more effective training pairs in kinship pairs (Zhou et al., 2019), but it is a time-consuming and labor-intensive process to construct a more suitable quadruple. In these works (Rehman et al., 2019; Nguyen et al., 2020; Yu et al., 2020), the dual-path structure network with shared or unshared weights is used to extract the face features, and various splicing methods are introduced for feature fusion, then the distance between the features is used for kinship verification. Recently, some works have started to focus on modifying the measurement method. In Wei et al. (2019), the traditional Euclidean distance or Mahalanobis distance measurement method is replaced by the adversarial learning method. In Wu et al. (2021), the framework of Mahalanobis remote metric learning is used to learn multiple distances from training data metrics. In Zhu et al. (2022), Distance and Direction based Deep Discriminant Metric Learning (D4ML) modifies and designs two loss functions to learn multiple metrics by making full use of the discriminative information contained in facial images of parents and children for minimizing the distance between relatives' faces. In conclusion, how to extract more effective shared features between kinship faces and learn the relationship between metrics is still a great challenge for metric learning-based kinship verification.

## Methodology

In section Motivation, the motivation behind the proposed method is detailed. In section Proposed architecture, the overall structure of the proposed network is described. In section Attention center learning guided multi-head attention mechanism, the proposed novel attention center learning guided multi-head attention mechanism is detailed. In section Family-level multi-center loss, the introduced kinship relation compare module is illustrated, and in section Relation compare module, the novel family-level multi-center loss is elaborated.

### Motivation

Multiple attention modules are introduced to extract local features under different channels in existing studies, however, the relationship between different attention modules is ignored, resulting in the inability to learn feature variability under different channels. To tackle this issue, we propose to conduct grouping at the channel level of the feature map, and then input it into a well-designed multi-headed attention module to extract local features of the face. The human brain, when processing visual information, is able to quickly focus on a few salient visual objects or multiple features, allowing for a broader range of visual information, whereas computer image processing is concerned with only a small fraction of the entire image. Therefore, to ensure the difference in various features to obtain a wider range of facial information as the human brain, we design the attention center learning module. The module is used to supervise the multi-head attention to learn diverse local features from different local regions.

Simply aggregating facial features of all family members to a single center generates an improper intra/inter-class distance, for example: inter-distance between husband and wife is closer than their intra-class distance, which leads to a wrong verification of kinship. The visual system of the human brain deals with the features of different individuals separately. Inspired by this reasoning, we believe that it is inappropriate to treat different members of the whole family as the same label for metric learning. Hence, we introduce a brain-inspired family-level multi-center loss so that the family feature center is not limited to one, and it is more useful to use the local area of the face to perform auxiliary metric learning.

In kinship verification, feature distance measurement is quite different and more challenging due to the existing data deficiency, gender difference, age gap, skin color, etc. Narrowing the intra-class distance with a general hand-designed metric function may not yield promising results. To handle the validation problem across multi-samples like the human brain, we propose to use a relational reasoning-based method to measure the similarity between relatives, instead of being limited to a manually set measurement function. Therefore, a non-linear relation comparable module is introduced to make the distance measurement more suitable for kinship verification.

### Proposed architecture

**Figure 2:**
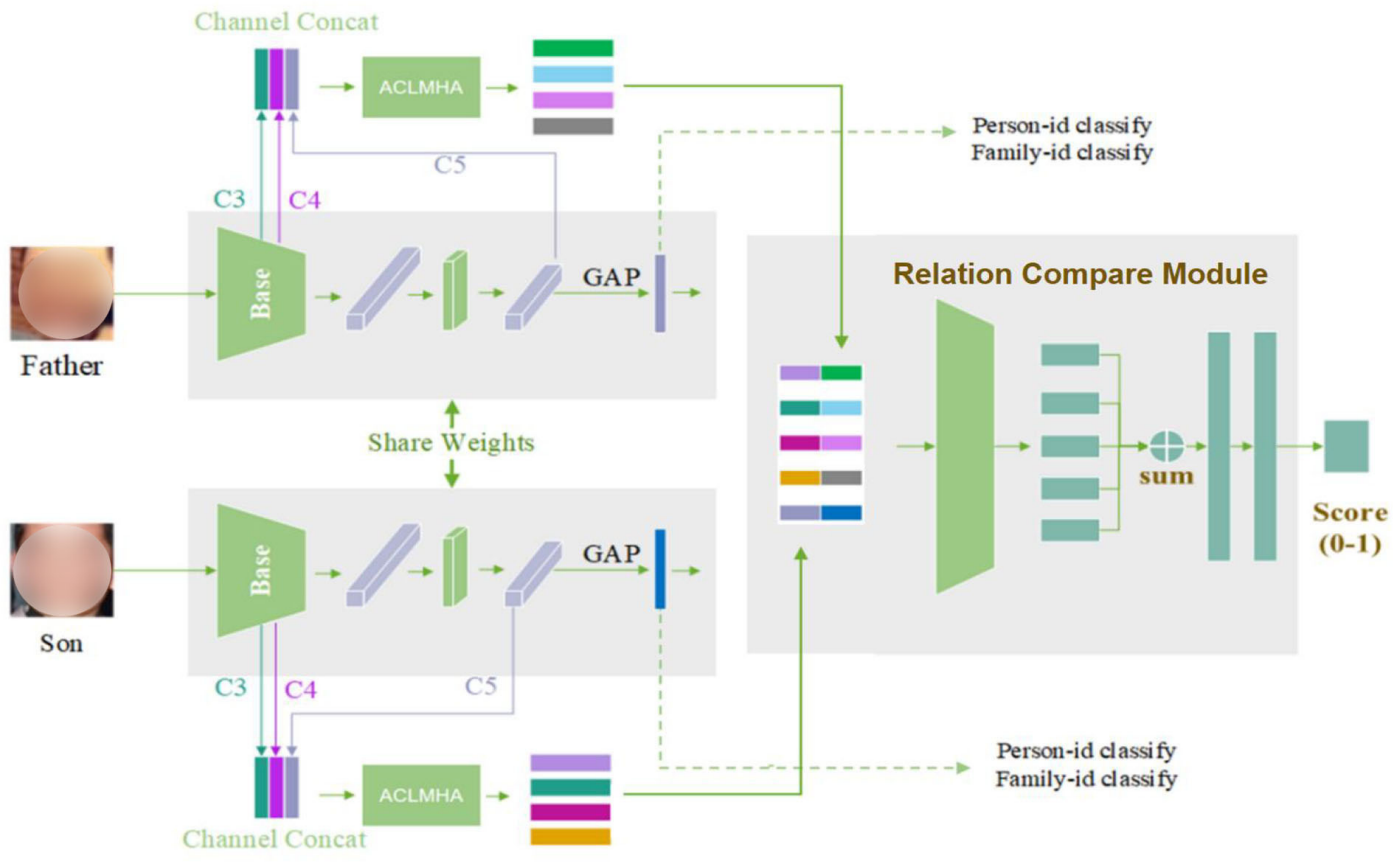
Overview of the kinship network structure.

In this section, we describe the proposed kinship verification framework. The overall structure, which consists of three main parts, is shown in Figure 2, in which the two input images are example images from FIW dataset (Robinson et al., 2018).

As shown above, the Siamese network is introduced as a feature extraction network architecture. The first part is a BaseNet module used for global feature extraction. It adopts a ResNet-50 network. To train the BaseNet better, the large-scale face dataset CASIA-WebFace (Yi et al., 2014) is introduced, and both SoftMax and center loss are applied. The second part is the attention center learning guided multi-head attention mechanism, also denoted as ACLMHA. It adopts the multi-head attention module to generate the local attention features of the face, during which, the proposed attention center learning mechanism is used to supervise the attention matrix. This helps to boost the deep model to capture various and abundant local features from different local face regions, and therefore improve the network's feature extraction ability for local areas. To capture small-scale local features better, we make full use of the feature maps (conv3_x, conv4_x, conv5_x) output by three different convolution blocks of ResNet, specifically, we perform bilinear upsampling operation for C5, use 1*1 convolution for down-channel for C4, and downsample for C3. Finally, three feature maps with a size of 14*14*512 are obtained., and then we get a 14*14*1,536 feature map after splicing in the channel layer, which is input into the ACLMHA model to extract the features of the local module. Besides, the brain-inspired family-level multi-center loss is proposed to address the feature distance expression further. The third part is the relation compare module introduced to measure the complex feature distance for kinship verification. The features obtained by splicing the global and local features of different faces are fed into the module to measure their similarity, and finally the kinship between face pairs is arrived at.

### Attention center learning guided multi-head attention mechanism

Attention module has been widely discussed to simulate the critical areas discovering process of the human brain. The non-local attention network expresses the importance of each pixel in the feature map through an autocorrelation matrix calculated by the correlation between the pixels of the feature map. To boost the attention module to focus on different critical regions of face images as human brain further, we propose an attention center learning mechanism to supervise the learned attention matrix. It guides the multi-head model to pay attention to different local features of the face image.

#### Structure of the ACLMHA

**Figure 3:**
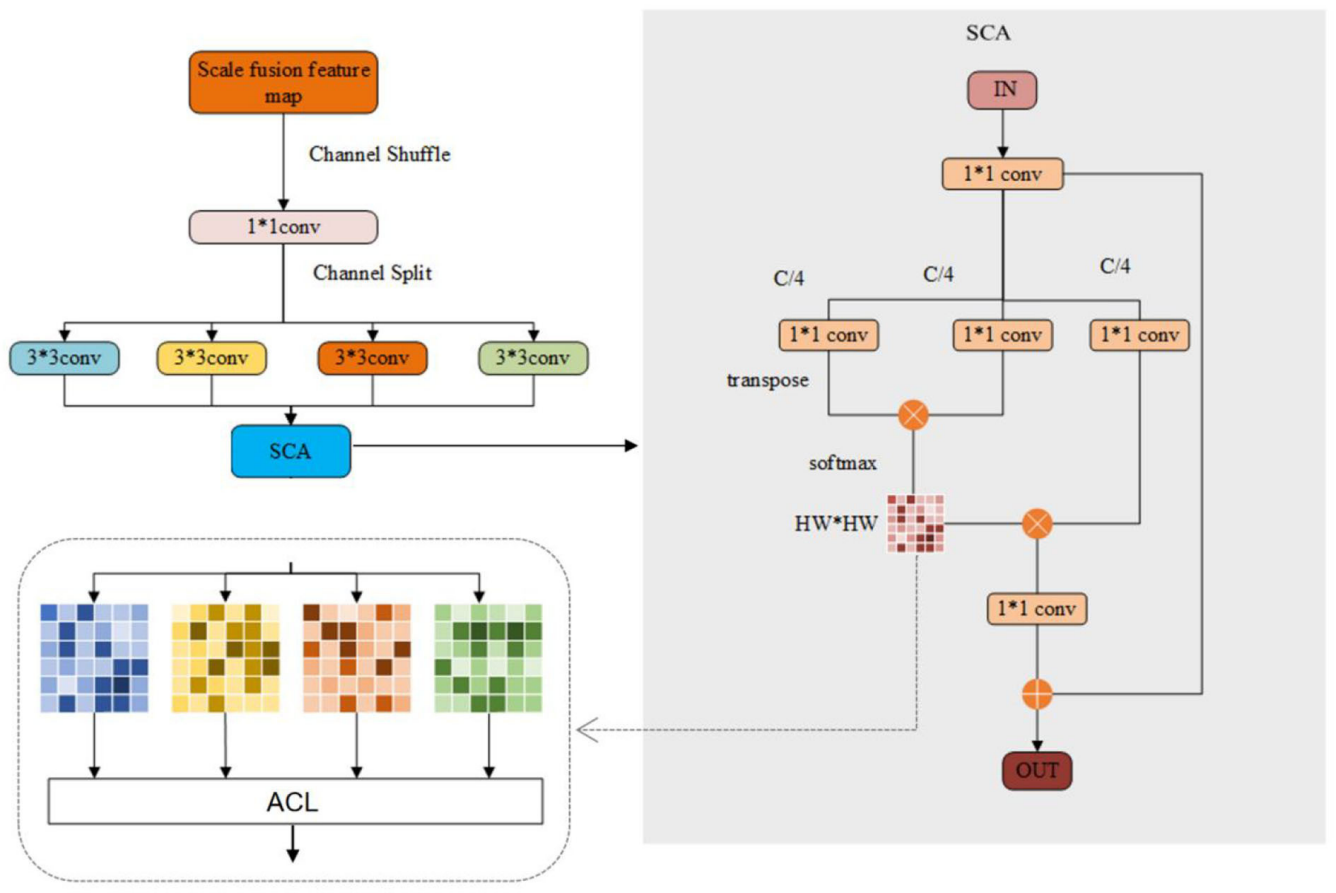
Overview of the structure of the ACLMHA (attention center learning guided multi-head attention mechanism).

As shown in Figure 3, considering that different channels often learn different visual modes of the image, we propose to extract the features of different regions by performing channel grouping on the convolved feature maps. To be specific, the feature maps of different scales obtained by the deep convolutional network are combined, and then channel shuffling is applied to mix the channel maps of different scales, so that the information of different scales can be merged. After that, the mixed combined features are divided into *k* groups, and subsequently convolution operations are performed on the features through *k* different convolutional layers. In this paper, *k* is set as 4. Then, the output is fed into the spatial-channel attention (SCA) network for spatial attention learning, during which the proposed attention center learning mechanism is applied to supervise the learned attention matrix to focus on different critical regions of face images as the human brain.

The SCA network can be represented as a triple (***K, Q, V***), as shown in Equation (1):

(1) $$\begin{array}{l} K^{i}\;=\;\theta \left(M^{i}\right),Q^{i}\;=\;\phi \left(M^{i}\right),V^{i}=\psi \left(M^{i}\right) \end{array}$$

where ***M*** ∈ *R*^*H* × *W* × *C*^ is the feature map fed to the attention module, *H, W, C* are the width, height, and channel of the feature map separately. *θ*,*ϕ*,*ψ* are three different 1 × 1 convolution layers. We use 1 × 1 convolution kernel to reduce the channel number $C\;to\;\frac{C}{m}$. In this paper, *m* = 4, the feature map ***K***^***i***^ is reshaped into $R\left(K^{i}\right)\in {\mathbb{R}}^{HW\times \frac{C}{m}}$ after passing through the 1*1 convolutional layer, the feature map ***Q*** is reshaped into $R{\left(Q^{i}\right)}^{T}\in {\mathbb{R}\;}^{\frac{C}{m}\times HW}$ after passing through the 1*1 convolutional layer and then transposed further. The autocorrelation matrix is obtained by multiplying ***K***^***i***^ and ***Q***^***i***^, and then the Softmax is performed row by row to get the final patch location attention matrix ***A***^***i***^ which is represented as shown in Equation (2):

(2) $$\begin{array}{l} A^{i}\;=\;SoftMax\left(R\left(K^{i}\right)\;•\;R{\left(Q^{i}\right)}^{T}\right) \end{array}$$

where ***R*** is the reshape operation, ***T*** is the transpose operation, and • represents the matrix multiplication operation. Then ***A***^***i***^ and ***R*****(*****V***^***i***^**)** are matrix multiplied and the residual ***M***^**′**^ is added to obtain the final local attention feature $M_{a}^{i}$, as shown in Equation (3):

(3) $$\begin{array}{l} M_{a}^{i}\;=\;R\left(V^{i}\right)\;•\;A^{i}+M^{\prime } \end{array}$$

#### Attention center learning module

The combination of different local features and global features of human faces has significant advantages over only global features. However, in most of the previous methods, features of different regions are extracted through facial landmark detection-based region location, which is not suitable for relatives, since the local similarities among relatives' faces are not limited to specific landmarks. Therefore, we developed an attention model that can automatically locate the salient areas between relatives, so that different attention matrices can learn different regions. We propose a feature center-based learning method to supervise the non-local attention correlation matrix.

**Figure 4:**
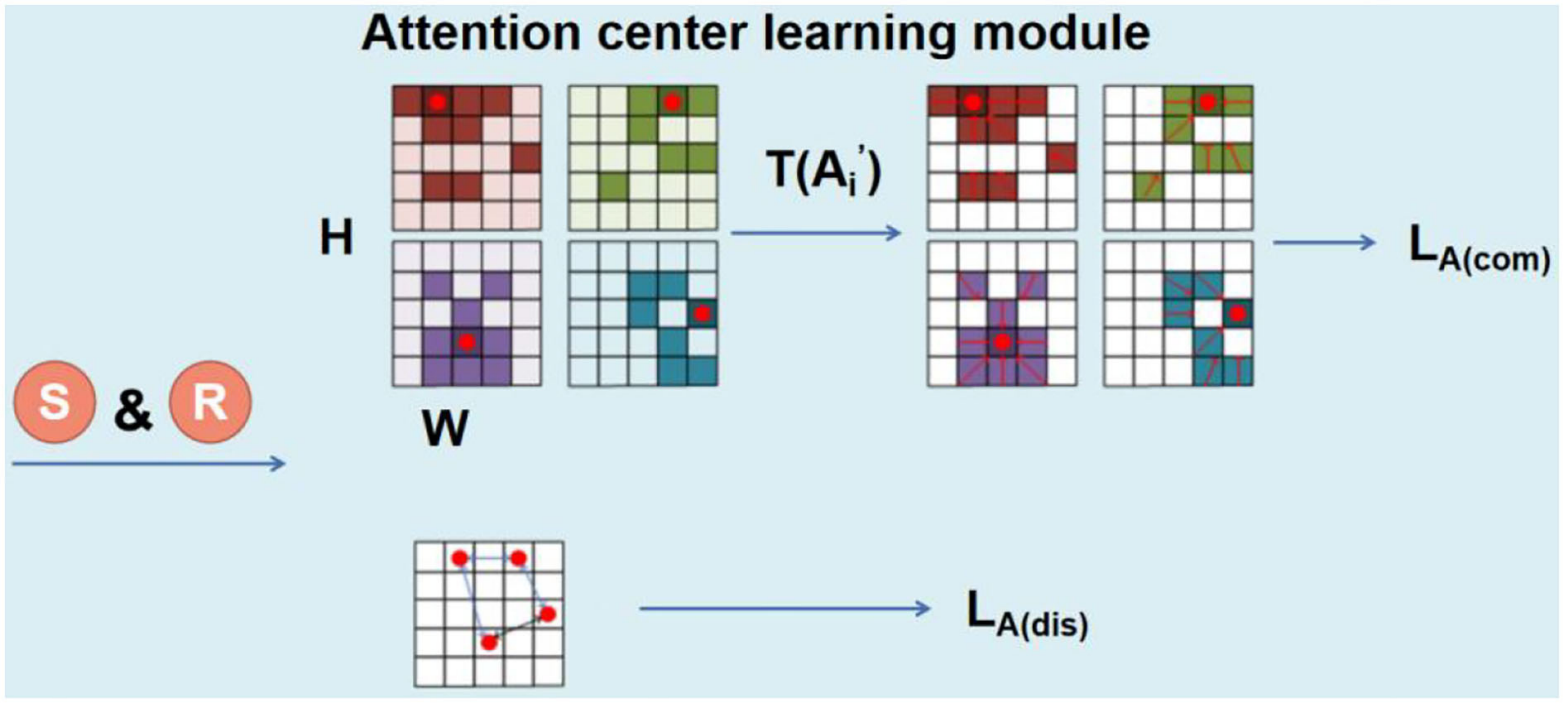
Overview of the structure of the attention center learning (ACL).

As shown in Figure 4, the location attention matrix is the result processed by the SoftMax function row-wise. We denote the operation of summing ***A***^***i***^ by column as ***S***, and then reshape it into ${A^{\prime }}_{i}\in {\mathbb{R}}^{H\times W}$, as shown in Equation (4):

(4) $$\begin{array}{l} {A^{\prime }}_{i}\;=\;R\left(S\left(A_{l}^{i}\right)\right) \end{array}$$

The maximum value of *A*^*i*^ after threshold operation is set as the center of the attention matrix, which is expressed in Equations (5)–(7):

(5) $$\begin{array}{l} {A^{\prime \prime }}_{i}\;=\;T\left({A^{\prime }}_{i}\right) \end{array}$$

(6) $$\begin{array}{l} T_{\theta }\left(a\right)=\{\begin{array}{cc} a_{i} & if\;a_{i}\geq \theta \\ 0 & otherwise \end{array} \end{array}$$

(7) $$\begin{array}{l} CenterA_{i}=\max\left({A^{\prime \prime }}_{i}\;\right) \end{array}$$

where ***T*** is the threshold operation to make sure only the area with larger attention weight is kept. To make $\theta \in \left(0,1\right)\;{A^{\prime }}_{i}$ is normalized.

To gather attention to the center, we proposed the ***L***_***A*****(*****com*****)**_. We introduce the reciprocal of the distance between each pixel in the matrix and the center is introduced to weight the point-to-point attention matrix value difference. So that pixels far from the center have smaller weights, and pixels closer to the center have larger weights. At the same time, for those pixels near the center point, their attention values are more close to that of the center point. ***L***_***A*****(*****com*****)**_ is represented as Equations (8) and (9):

(8) $$\begin{array}{l} L_{A\left(com\right)}\;=\;\\ \overset{H}{\underset{x\;=\;0}{\sum }}\overset{W}{\underset{x\;=\;y}{\sum }}\left(\frac{1}{||\left(x,y\right)-\left(C_{x},C_{y}\right)|{|}_{2}^{2}}\;•\;||{A^{\prime \prime }}_{i}\left(C_{x},C_{y}\right)-{A^{\prime \prime }}_{i}\left(x,y\right)|{|}_{2}^{2}\right) \end{array}$$

(9) $$\begin{array}{l} and\;{A^{\prime \prime }}_{i}\left(x,y\right)\neq 0 \end{array}$$

The purpose of ***L***_***A*****(*****dis*****)**_ is to separate the attention centers from each other, which helps to extract the attention feature maps of different positions on the face. ***L***_***A*****(*****dis*****)**_ is represented as Equation (10):

(10) $$\begin{array}{l} L_{A\left(dis\right)}\;=\;-\overset{k}{\underset{i\;=\;0}{\sum }}\overset{k}{\underset{j\neq i}{\sum }}||\left(C_{x}^{i},C_{y}^{i}\right),\left(C_{x}^{j},C_{y}^{j}\right)|{|}_{2}^{2} \end{array}$$

Finally, the loss function **L**_**A**_ of the ALMACL part is obtained by adding the above two loss functions, as shown in Equation (11).

(11) $$\begin{array}{l} L_{A}\;=\;L_{A\left(com\right)}+L_{A\left(dis\right)} \end{array}$$

### Family-level multi-center loss

**Figure 5:**
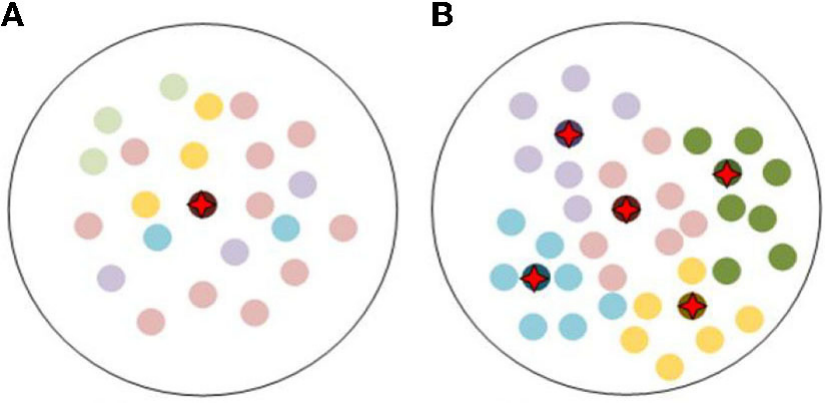
To understand the distribution of facial features in the feature space, the figure on the left shows the case of a single center **(A)**, each light color dot represents different family members' face samples, and the dark dot with the star represents the characteristics of the family id center, and the figure on the right shows the distribution of features with multiple centers **(B)**.

Different from the previous kinship verification method, in this paper we propose a family-level multi-center loss, which is a combination of the SoftMax function and the designed multi-center loss. Inspired by SoftTriple loss (Qian et al., 2019), as shown in Figure 5A, simply mapping the feature of father, mother, and child to the same feature center is improper, because, although children have latent similarities with their parents, fathers and mothers do not have such similarities. Single feature center will lead to improper intra/inter-class distance for kinship verification. To combat this issue, we design multiple feature centers for each family label, which we call family-level multi-center loss. As shown in Figure 5B, the features of different family members can be aggregated to the nearest center point by extending out multiple centers, which helps to separate the feature boundaries of different members. The family-level multi-center loss function ***L***_***fid*****−*****c***_ is specified by the following Equation (12):

(12) $$\begin{array}{l} L_{fid-c}\;=\;\frac{1}{2Nm}\overset{N}{\underset{i\;=\;1}{\sum }}\overset{m}{\underset{k\;=\;1}{\sum }}||\alpha_{i}-c_{\beta_{i}}^{k}|{|}_{2}^{2} \end{array}$$

where *N* is the number of samples in each minibatch, *m* is the number of each family center, and ${\text{c}}_{\beta_{\text{i}}}^{\text{k}}$ is the category center, and the updated equations of the category center are as follows:

(13) $$\begin{array}{l} \frac{\partial Lfid-c}{\partial \alpha_{i}}\;=\;\frac{1}{Nm}\overset{m}{\underset{k\;=\;1}{\sum }}\left(\alpha_{i}-c_{\beta_{i}}^{k}\right) \end{array}$$

(14) $$\begin{array}{l} \Delta C_{j}\;=\;\frac{\sum_{i\;=\;1}^{N}\delta_{\left(\beta_{i}\;=\;j\right)}\left(c_{j}-\alpha_{i}\right)}{\varepsilon +\sum_{i\;=\;1}^{N}\delta_{\left(\beta_{i}\;=\;j\right)}} \end{array}$$

(15) $$\begin{array}{l} C^{\prime }\;=\;C_{j}-a\Delta C_{j} \end{array}$$

where *α*_***i***_ denotes the sample, *β*_***i***_ denotes the corresponding label, ε is used to prevent the denominator from being zero when updating the calculation of categories with multiple feature centers, and δ() indicates that the value corresponding to the sample in the current training batch is 1 and the value corresponding to other samples is 0.

As shown in Figure 2, to ensure that the effective face features can be extracted, we propose a classification loss function ***L***_***cls***_, which includes two parts: ***L***_***fid*****−*****CE***_ and ***L***_***pid*****−*****CE***_, as shown in Equations (16) and (17), and the total loss is shown in Equation (18).

(16) $$\begin{array}{l} L_{fid}\;=\;L_{fid-CE}+L_{fid-c} \end{array}$$

(17) $$\begin{array}{l} L_{cls}\;=\;\lambda L_{fid-CE}+\beta L_{pid-CE} \end{array}$$

(18) $$\begin{array}{l} L_{total}\;=\;L_{cls}+L_{A}+L_{fid} \end{array}$$

### Relation compare module

As shown in Figure 2, 2*K groups of facial local features extracted from the input image pair are combined to generate K*K features and then spliced with the global features. The obtained final features are fed into the perceptron layer, followed by an element-level addition operation, the output of which is used for family relationship learning. Finally, the kinship/non-kinship score of the face pair are acquired through the sigmoid activation function, as shown in Equation (19).

(19) $$\begin{array}{l} score\;=\;g(sum\left(f\left(cat\left(M_{a}^{i}\left(X_{1}\right),\;M_{a}^{i}\left(X_{2}\right),Z\left(X_{1}\right),Z\left(X_{2}\right)\right)\right)\right) \end{array}$$

Among them, ***X***_**1**_ represents the input image of the child, ***X***_**2**_ represents the input image of the parents, ***cat*** represents concatenation operation, and ***Z*** represents the mapping of the BaseNet network, which is used to extract global features. In addition, we use binary cross entropy (BCE) loss for training here. This module is similar to a learnable metric function. Through training, it can learn the feature relationships of faces among different family relationships. Therefore, it can overcome the limitations of hand-designed metric functions and learn the potential relationships between features better.

## Experimental results

### Datasets

**Figure 6:**
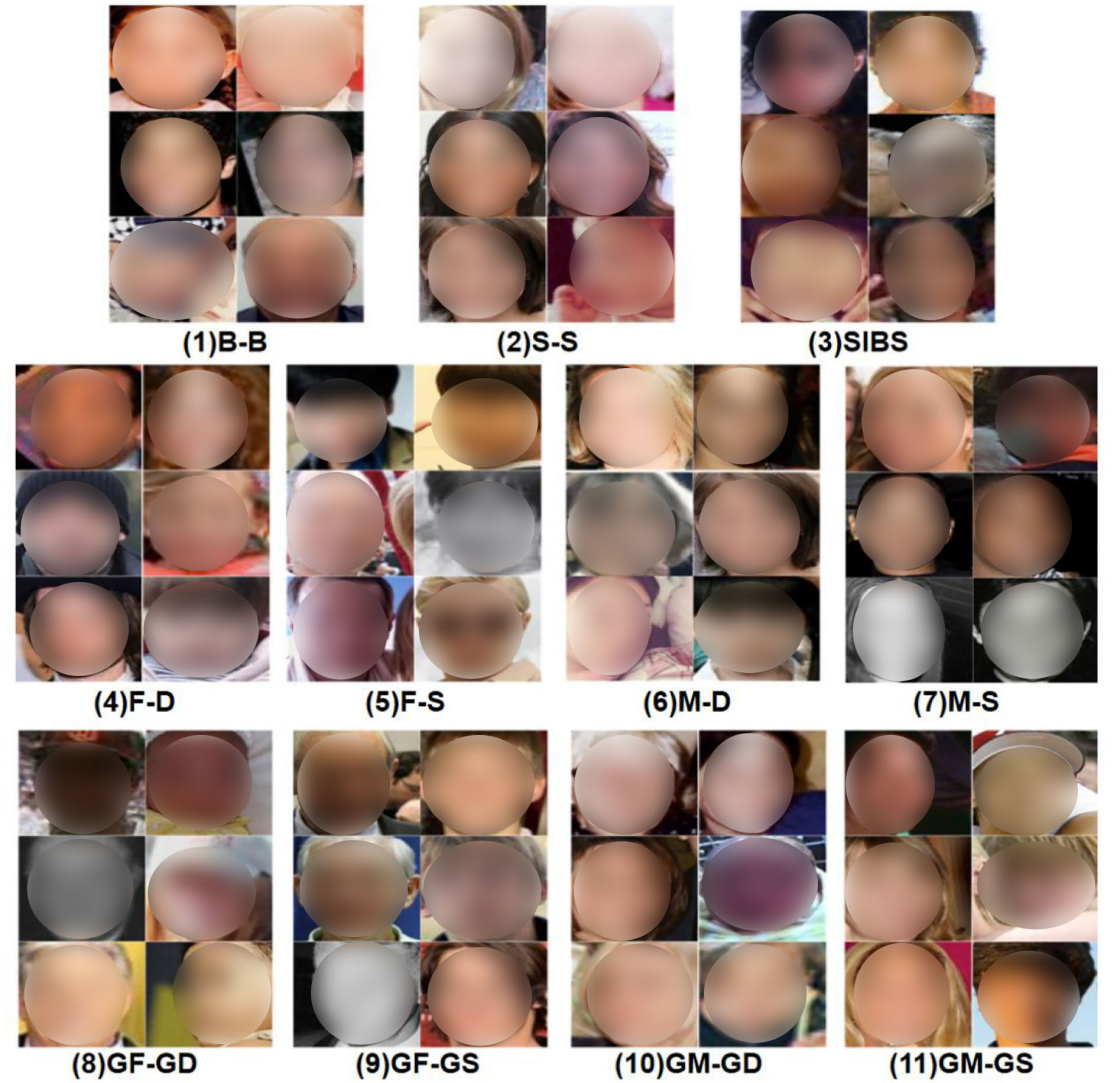
Examples of Family in the Wild (FIW) dataset (Robinson et al., 2018).

The face kinship verification Family in the Wild (FIW) dataset (Robinson et al., 2018) is adopted for experiments in this paper. FIW is the largest dataset whose distribution is closest to the real data. As shown in Figure 6, the dataset contains 1,000 families and 10,676 individuals. It can be formed into 690 thousand pairs, including all the 11 kinds of kinship: B-B, S-S, SIBS, F-D, F-S, M-D, M-S; GF-GD, GF-GS, GM-GD, and GM-GS.

### Training details

First, the CASIA-WebFace database is used to train the BaseNet, during which the combination of SoftMax and center loss is employed. We notice that, at the initial stage of training, if center loss is assigned with larger weight, it will lead to a very slow or difficult convergence. So, we propose to introduce a similar warm-up strategy that can dynamically adjust the weight of the center loss. Specifically, we start with a relatively small weight at the beginning of the training stage. In this paper, we set it to 0.5. After 200 thousand iterations, the weight of every five thousand iteration is 1.5 times of the original, until the iteration is completed. Second, we migrate the pretrained BaseNet on FIW. Only the last fully connected layer is left and all the parameters are frozen to learn the subsequent kinship model with a small learning rate. When the network is iterated to 200 thousand times, we unfreeze all the network layers and then fine-tune the entire network. Finally, in the verification phase, two face images are input into our model to verify their kinship.

### Ablation experiments

To explore the effectiveness of our relationship model for latent feature learning among facial relatives, we design a group of comparative experiments between the multi-layer perceptron model (MLP), the relation compare model (RCM), and the relation compare module combined with ACLMHA. It should be noted that for the first two methods, the adopted features are the combination of the global and local features, which are extracted through MTCNN (Multi-task Cascaded Convolutional Networks)-based key points detection.

As shown in Table 1, the verification accuracy of RCM is increased by about 7% compared to the traditional MLP. In addition, ACLMHA combined with RCM achieves the highest result, which is a further 2% improvement. It shows that the proposed ACLMHA can enhance further the discrimination of local features compared to those. In addition, the average accuracy of each generation is 80.7, 78.4, and 75.3% separately, which shows that kinship verification of the second generation is the most challenging task.

**Table 1:** Ablation study results on family in the wild (FIW).

**Method**	**B-B**	**S-S**	**SIBS**	**F-D**	**F-S**	**M-D**	**M-S**	**GF-GD**	**GF-GS**	**GM-GD**	**GM-GS**	**Avg**
MTCNN + MLP	72.2	73.8	70.0	70.1	72.8	71.0	69.5	68.2	65.4	67.8	63.0	69.4
MTCNN + RCM	79.1	80.2	77.8	75.4	78.6	77.2	76.4	76.8	73.4	76.2	70.5	76.5
ACLMHA + RCM	81.3	82.1	78.6	77.3	80.5	78.4	77.4	78.5	74.1	76.4	72.2	77.5

### Comparative experiments

To demonstrate further the advantages of our algorithm, the proposed algorithm is compared with other advanced algorithms published so far, and the specific comparison results are shown as follows.

**Figure 7:**
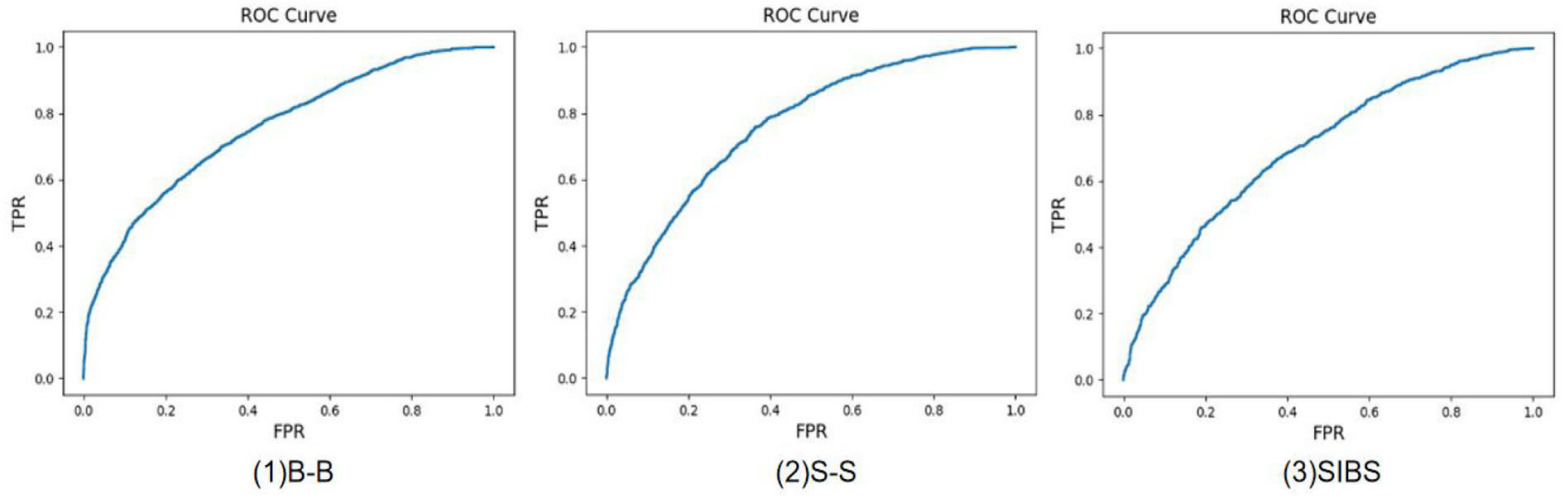
Receiver operating characteristic (ROC) curve of brother-brother (B-B), sister-sister (S-S), and brother-sister (SIBS).

**Figure 8:**
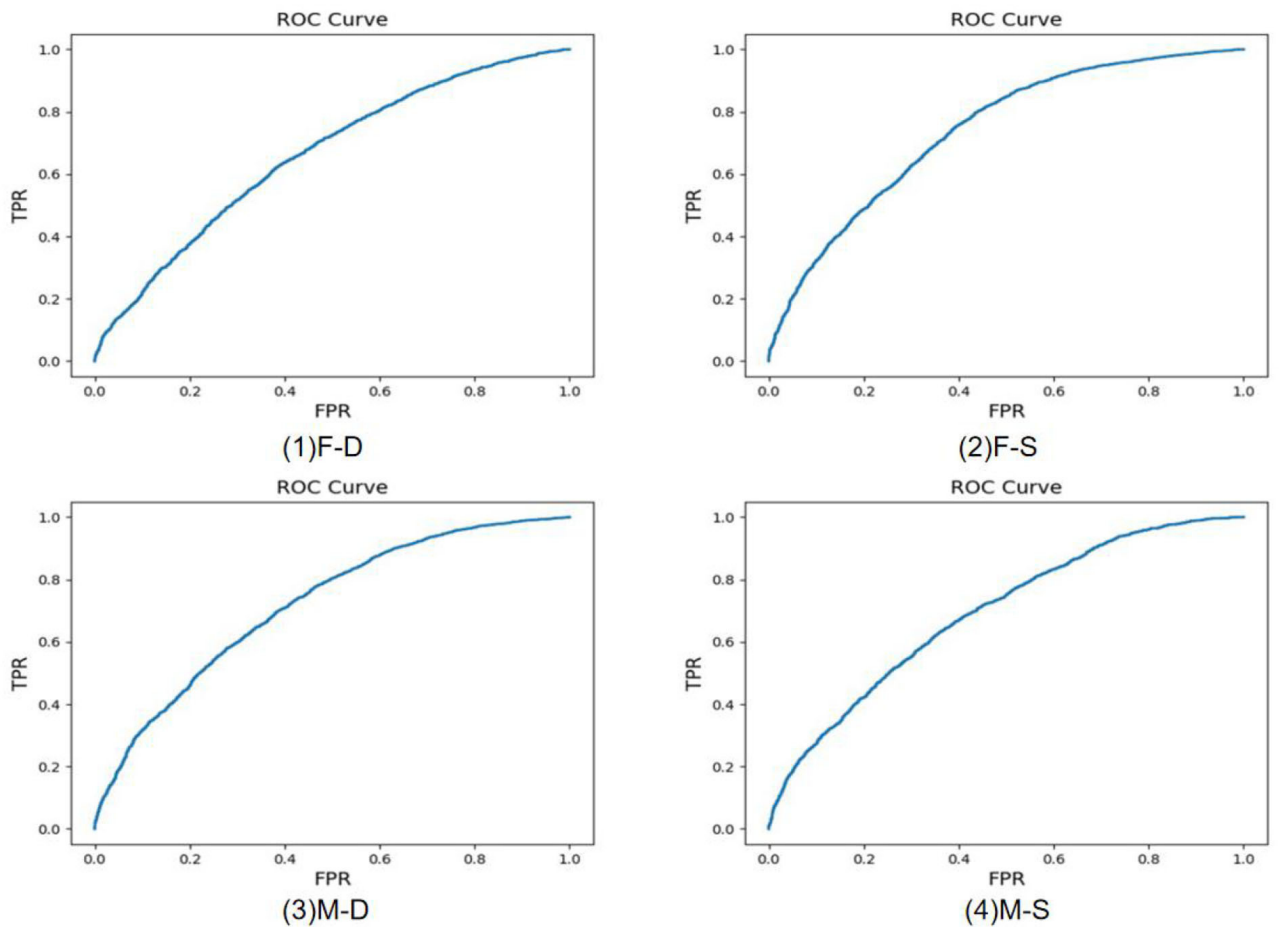
Receiver operating characteristic (ROC) curve of father-daughter (F-D), father-son (F-S), mother-daughter (M-D), and mother-son (M-S).

**Figure 9:**
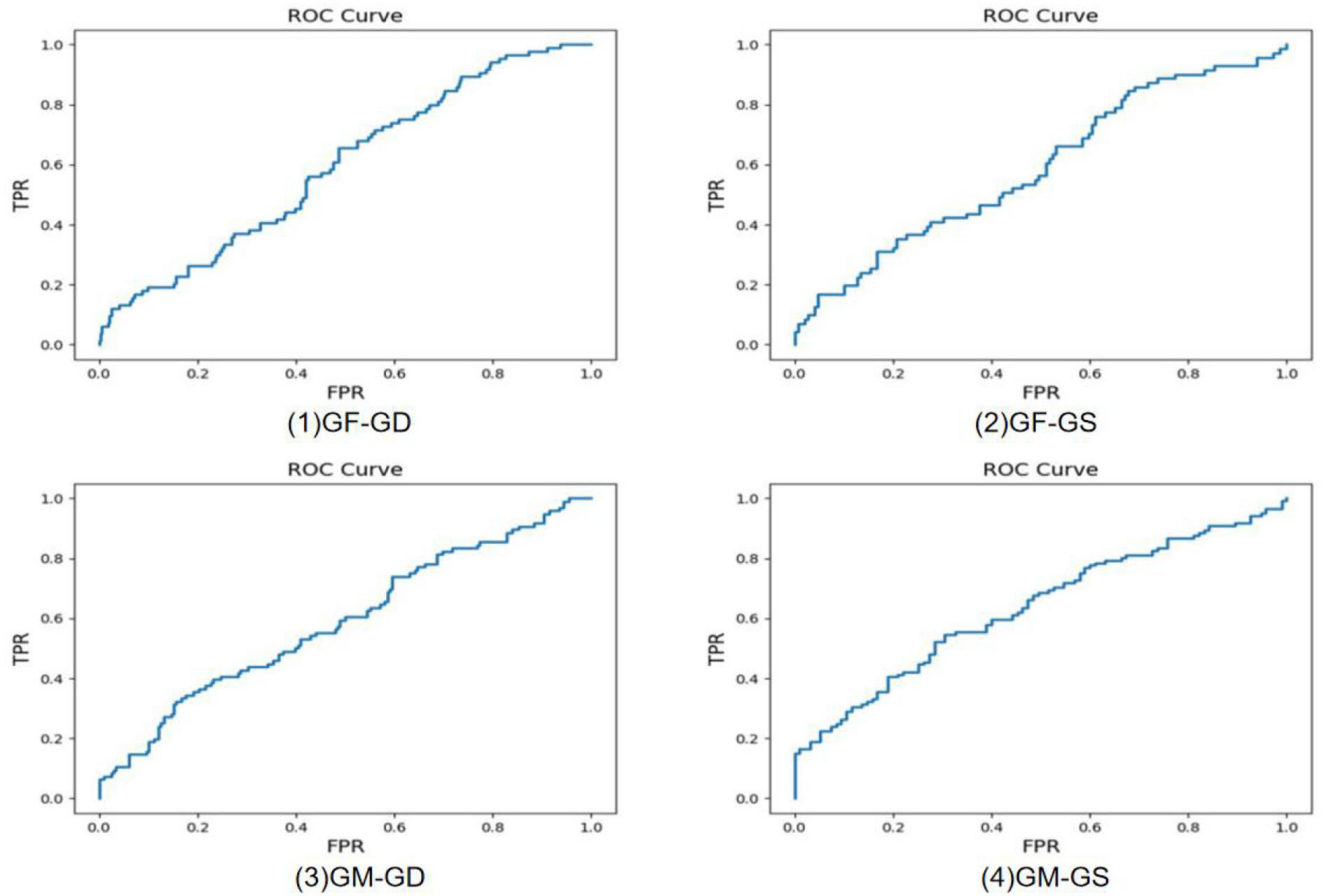
Receiver operating characteristic (ROC) curve of grandfather-granddaughter (GF-GD), grandfather-grandson (GF-GS), grandmother-granddaughter (GM-GD), and grandmother-grandson (GM-GS).

Experiments of 11 different kinship verifications are conducted. Figures 7–9 show the receiver operating characteristic (ROC) curve of the proposed method on the FIW dataset. The higher the AUC (area under curve) value, the higher the prediction accuracy. It can be seen that the same-generation kinship verification achieves the best effect, followed by the first-generation kinship. The second-generation kinship verification is the most challenging task.

Tables 2–4 show the comparison results of the proposed method and the current state-of-the-art (SOTA) methods. As shown, for the proposed method, the average verification accuracy of the same-generation is 80.7%, which is 1.4% higher than the best results of other comparable algorithms. The average accuracy of the first-generation kinship is 78.4%, during which the M-D kinship verification achieves the highest result among all the mentioned methods. The average accuracy of the most challenging second-generation kinship is 75.3%, which is 3.5% higher than the best results of other algorithms, which however achieves much higher performance compared with other optimal algorithms. The results show that the kinship verification evaluation of our method shows a greater improvement compared to the existing algorithm, especially for the most challenging second-generation kinship verification task.

**Table 2:** Results of brother-brother (B-B), sister-sister (S-S), and brother-sister (SIBS) on family in the wild (FIW).

**Method**	**B-B**	**S-S**	**SIBS**	**Avg**
LBP (Ahonen et al., 2006)	55.5	57.5	55.4	56.1
SIFT (Dalal and Triggs, 2005)	57.9	59.3	56.9	58.0
VGG-face (Parkhi et al., 2015)	69.7	75.4	66.5	70.5
ResNet20 (Wen et al., 2016)	65.6	69.7	60.1	65.1
SphereFace (Liu et al., 2017)	71.9	77.3	70.2	73.1
ResNet50 (Hörmann et al., 2020)	66.4	65.3	76.0	69.2
ResNet50 + feature fusion (Yu et al., 2020)	75.1	74.4	72.0	73.8
InsightFace (Shadrikov, 2020)	80.2	80.4	77.3	79.3
Dual-VGGFace-v2 (Rachmadi et al., 2021)	66.3	73.2	67.2	68.9
AIAF + IFW (Liu et al., 2022)	73.8	85.5	77.6	78.9
Ours	81.3	82.1	78.6	80.7

**Table 3:** Results of father-daughter (F-D), father-son (F-S), mother-daughter (M-D), and mother-son (M-S) on family in the wild (FIW).

**Method**	**F-D**	**F-S**	**M-D**	**M-S**	**Avg**
LBP (Ahonen et al., 2006)	55.1	53.8	55.7	54.7	54.8
SIFT (Dalal and Triggs, 2005)	56.4	56.2	55.1	56.5	56.1
VGG-face (Parkhi et al., 2015)	64.3	63.9	66.4	62.8	64.4
ResNet20 (Wen et al., 2016)	59.5	60.3	61.5	59.4	60.2
SphereFace (Liu et al., 2017)	69.3	68.5	71.8	69.5	69.8
ResNet50 (Hörmann et al., 2020)	76.9	80.1	76.7	78.2	78.0
ResNet50 + feature fusion (Yu et al., 2020)	75.5	81.8	74.7	75.2	76.8
InsightFace (Shadrikov, 2020)	75.2	80.8	77.7	74.4	77.0
Dual-VGGFace-v2 (Rachmadi et al., 2021)	65.3	64.1	67.3	66.3	65.8
AIAF + IFW (Liu et al., 2022)	79.1	78.2	76,1	86.5	79.9
Ours	77.3	80.5	78.4	77.4	78.4

**Table 4:** Results of grandfather-granddaughter (GF-GD), grandfather-grandson (GF-GS), grandmother-granddaughter (GM-GD), and grandmother-grandson (GM-GS) on family in the wild (fiw).

**Method**	**GF-GD**	**GF-GS**	**GM-GD**	**GM-GS**	**Avg**
LBP (Ahonen et al., 2006)	55.8	55.9	54.0	55.4	55.3
SIFT (Dalal and Triggs, 2005)	57.3	55.4	57.3	56.7	56.7
VGG-face (Parkhi et al., 2015)	62.1	63.8	57.4	61.6	61.2
ResNet20 (Wen et al., 2016)	55.4	58.1	59.7	59.7	58.2
SphereFace (Liu et al., 2017)	66.1	66.4	64.6	65.4	65.6
ResNet50 (Hörmann et al., 2020)	70.0	73.4	63.9	60.3	66.9
ResNet50 + feature fusion (Yu et al., 2020)	72.5	72.7	67.3	67.6	70.0
InsightFace (Shadrikov, 2020)	77.9	69.4	75.8	59.8	70.7
Dual-VGGFace-v2 (Rachmadi et al., 2021)	60.5	59.1	61.6	60.6	60.5
AIAF + IFW (Liu et al., 2022)	69.3	69.3	70.5	78.3	71.8
Ours	78.5	74.1	76.4	72.2	75.3

## Conclusions

In this paper, we propose a novel brain-inspired network with ACLMHA and FML to address the challenging feature expression, complex similarity measurement issues, and the misclassification due to single feature center in kinship verification. First, we propose an attention center learning guided multi-head attention mechanism to supervise the learning of attention weights and make different attention heads notice the characteristics of different regions to boost the deep model to capture various and abundant local features from different local face regions. Second, a family-level multi-center loss is proposed to ensure that the learned model can map different facial features of the same family to similar positions in the feature space. Finally, the feature relation compare module is introduced to measure the potential similarity of features among relatives. Extensive comparison experiments are conducted on the FIW dataset. Among them, the proposed method achieves a promising performance, especially in the verification of grandparents and grandchildren, which is significantly better than other state-of-art (SOTA) methods. The topic of how to combat data scarcity and better utilize the existing face dataset to improve the accuracy of facial kinship verification needs to be discussed in the future.

## Data availability statement

Publicly available datasets were analyzed in this study. This data can be found here: http://smile-fiw.weebly.com/.

## Author contributions

CL and MB: methodology, software, writing—original draft, review, and editing. LZ: methodology, software, writing—original draft, and review. KX: project administration, supervision, and conceptualization. WS: investigation, validation, and supervision. HZ: writing—review and formal analysis. All authors contributed to the article and approved the submitted version.
